# Clinical utility of circulating tumor cells: an update

**DOI:** 10.1002/1878-0261.12869

**Published:** 2020-12-25

**Authors:** Antoine Vasseur, Nicolas Kiavue, François‐Clément Bidard, Jean‐Yves Pierga, Luc Cabel

**Affiliations:** ^1^ Department of Medical Oncology Paris and Saint‐Cloud Institut Curie France; ^2^ UVSQ Paris‐Saclay University France; ^3^ Circulating Tumor Biomarkers laboratory Inserm CIC‐BT 1428 Institut Curie Paris France; ^4^ Paris University France

**Keywords:** circulating tumor cells, clinical utility, clinical validity, CTC‐derived xenografts, liquid biopsy

## Abstract

The prognostic role of circulating tumor cells (CTCs) has been clearly demonstrated in many types of cancer. However, their roles in diagnostic and treatment strategies remain to be defined. In this review, we present an overview of the current clinical validity of CTCs in nonmetastatic and metastatic cancer, and the main studies or concepts investigating the clinical utility of CTCs. In particular, we focus on breast, lung, colorectal, and prostate cancer. Two major topics concerning the clinical utility of CTC are discussed: treatment based on CTC count or CTC variations, and treatment based on the molecular characteristics of CTCs. Although some of these studies are inconclusive, many are still ongoing, and their results could help to define the role of CTCs in the management of cancers. A summary of published or ongoing phase II‐III trials is also presented.

## Graphical abstract

This review article discusses the clinical utility of circulating tumor cells (CTCs) in various settings of screening and treatment of non‐metastatic and metastatic tumors. Two major aspects are considered, namely i) treatment based on CTC count or CTC variations, and ii) treatment based on the molecular characteristics of CTCs. A summary of published or ongoing phase II‐III trials with CTCs is also presented.

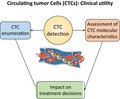

**Abbreviations**
AR: androgen receptor
BC: breast cancer
CTCs: circulating tumor cells
ctDNA: circulating tumor DNA
CRC: colorectal cancer
CRPC: castration‐resistant prostate cancer
CDX: CTC‐derived xenografts
tCTC: CTC trajectory
DFS: disease‐free survival
DDFS: distant disease‐free survival
FISH: fluorescent in situ hybridization
ICI: immunocheckpoint inhibitors
IHC: immunohistochemistry
ITH: intratumor heterogeneity
ISET: Isolation by Size of Epithelial Tumor Cells
NCT: neoadjuvant chemotherapy
NSCLC: nonsmall cell lung cancer
OS: overall survival
PFS: progression‐free survival
PSMA: prostate‐specific membrane antigen
PV: pulmonary vein
SCLC: small cell lung cancer

## 1 Introduction

Circulating tumor cells (CTCs) are cells that have been detached from the primary tumor or metastases and entered the circulation. CTCs can cause metastases in various organs by passing through the bloodstream [1]. First described more than 150 years ago [2], CTCs are rare in blood, but can now be detected and counted following their separation from blood cells using various enrichment methods, reviewed elsewhere [3]. CellSearch^®^, the only FDA‐approved technology for CTC isolation [4], involves selection of EpCAM‐expressing cells with antibody‐labeled magnetic nanoparticles, and detection cytokeratin‐ and DAPI‐positive, CD45‐negative cells by fluorescent microscopy [5].

A high CTC count has been associated with poor prognosis in several cancers and at various disease stages, especially in breast, lung, and prostate cancer. However, the clinical utility of the CTC count remains to be demonstrated. For instance, in metastatic breast cancer (BC), clinical practice guidelines currently do not recommend the use of CTCs for treatment decisions [6], while results from several CTC‐based interventional clinical trials are still pending.

In this review, we summarize the evidence that has established CTCs as an independent prognostic factor in several cancer types, in both localized and metastatic settings, with a particular focus on BC. We also review the clinical trials designed to demonstrate the clinical utility of CTCs and discuss potential applications of CTCs.

## 2 Clinical validity of CTCs

### 2.1 Cancer screening

Several studies have detected CTCs at early stages of tumor development [7, 8, 9, 10, 11, 12, 13]. CTC detection in a cancer screening setting could therefore enable early cancer detection and treatment. This approach has been studied notably in lung cancer: Ilie *et al*. reported that among 168 patients with chronic obstructive pulmonary disease undergoing annual surveillance spiral computer tomography for lung cancer, all 5 patients with a positive CTC count developed lung cancer during follow‐up. In this study, CTCs were detected one to four years earlier than radiological signs of malignancy on computed tomography [14]. In a second study, Fiorelli *et al*. used a filtration‐based detection technique, namely Isolation by Size of Epithelial Tumor Cells (ISET) [15], to assess the potential of CTC counts for differentiating benign from malignant lung lesions; with a CTC count cutoff of 25 cells [16], CTCs were detected in 90% of patients with malignant disease and 5% of patients with benign disease. CTCs could therefore also help to distinguish malignant from benign lesions and may constitute a valid biomarker for the diagnosis of lung cancer.

Based on these promising results with ISET technology, a multicenter prospective study using ISET in a cohort of patients eligible for lung cancer screening was conducted with three CTC screening rounds at one‐year intervals [17]. Among the 614 participants, 19 (3%) were diagnosed with a prevalent lung cancer at baseline and 19 were diagnosed with an incident lung cancer (15 (3%) of 533 patients at year one and 4 (1%) of 477 patients at year two). Extrapulmonary cancers were diagnosed in 27 (4%) participants. The sensitivity of baseline CTC detection for lung cancer detection was 26% (95% CI 12–49). ISET technology had a poor performance to predict the subsequent development of pulmonary or extrapulmonary cancers based on CTC counts, as only 2 of the 13 lung cancers and 0 of the 13 **(**10 at year one and 3 at year two) extrapulmonary cancers detected within 2 years had positive CTC detection at baseline. More sensitive assays must therefore be used in the future trials, as the sensitivity described here was unacceptable in a clinical screening setting [17].

In a context other than lung cancer, our group has initiated the CirCa 01 trial (NCT02608346), which includes deleterious *BRCA1* mutation carriers without evidence of cancer. The CirCa 01 trial will evaluate the sensitivity and specificity of detection of *TP53* mutations in the plasma (circulating tumor DNA‐ctDNA) for the diagnosis of relapse or new cancer growth. Within the CirCa 01 cohort of Institut Curie, some individuals will be invited to participate in a CTC substudy that will evaluate the screening performances of the CTC enumeration by the CellSearch^®^ system.

At this time, mainly due to low detection sensitivity, CTCs have not demonstrated clinical validity in the context of cancer screening.

### 2.2 Localized tumors

The CTC count is usually low in the nonmetastatic setting, as shown in the cancer screening section, therefore the CTC detection cutoff has often been set at ≥ 1 CTC/7.5 mL of blood in most studies (usually with the CellSearch^®^ system), while the CTC count in the metastatic setting is often ≥ 5 CTC/7.5 mL of blood, as described below.

#### 2.2.1 Breast cancer

The clinical validity of CTCs in early BC has been studied in both adjuvant and neoadjuvant treatment settings.

In a pooled analysis using CellSearch^®^ that included 3,173 patients with localized BC across five cancer centers [18], the percentage of patients with at least one CTC was 20%, and the presence of CTCs was an independent prognostic factor for disease‐free survival (DFS; HR = 1.8; 95% CI 1.5–2.3) and overall survival (OS; HR = 2; 95% CI, 1.5–2.6). The prognostic value of CTCs has also been observed in other studies [19, 20, 21, 22]. The CellSearch® system, which allows the morphological analysis of detected CTCs, was used in most of the largest studies, as there were initially some concerns that mRNA‐based techniques could be associated with false positives [23]. However, it should be noted that methods using a well‐defined threshold for reverse transcriptase PCR‐based techniques ultimately demonstrated good performances for CTC detection [24, 25]. The cutoff used for CTC positivity with the CellSearch^®^ system was ≥ 1 per blood sample. In some of the studies conducted in early BC [21, 22], CTC counts were assessed before and after adjuvant chemotherapy, and seemed to predict lower relapse‐free survival and overall survival at both time‐points, but this correlation was not significant for OS in the study by Rack *et al*. [21]. Although not consistently described in all previous trials of CTCs in localized BC [21, 26, 27], the pooled analysis by Janni *et al*. showed that the presence of CTCs was associated with higher histological grade, lymph node involvement and tumor size, and a lobular histology. Interestingly, in a large trial with 1,697 patients, CTC count was shown to possibly predict a benefit of radiotherapy in the adjuvant setting of early BC [28], as radiotherapy was associated with longer local recurrence‐free survival, disease‐free survival, and longer OS only in patients with detectable CTCs. During follow‐up of HER2‐negative stage II‐III BC, detection of CTCs (5.1% of patients) between 4.5 and 7.5 years after primary surgical treatment was associated with a 13.1‐fold higher risk of recurrence [29].

In a meta‐analysis of individual data from 21 studies (*n* = 1574) in the neoadjuvant setting, preneoadjuvant chemotherapy (NCT) and presurgery CTC counts were significantly associated with both worse OS and worse distant disease‐free survival (DDFS) [30]. In patients with one, two, three to four, and five or more CTCs before NCT, mortality HRs were 1.1 (95% CI = 0.65–1.7), 2.6 (95% CI = 1.4–4.5), 3.8 (95% CI = 2.1–6.7), and 6.3 (95% CI = 4.3–9.1), respectively. The pre‐NCT (but not presurgery) CTC count was also associated with locoregional relapse‐free survival. This meta‐analysis found a slightly higher pathological complete response rate in patients with no CTC detected before NCT compared to patients with at least one CTC detected, but this association was not significant for presurgery CTC or with any another CTC positivity cutoff, and multivariate analysis did not show any association between CTC detection at any time‐point and pathological complete response rate. There was an association between CTC count and tumor size, but it was largely driven by T4d tumors (inflammatory BC). An association between CTC detection before NCT and OS or DFS was also reported in most recent studies, which are extensively reviewed here [31].

#### 2.2.2 Gastrointestinal cancers

Various studies have demonstrated the prognostic value of CTCs for OS in localized colorectal cancer (CRC) [7, 13]. In a series of 287 patients, Bork *et al*. found that preoperative CTC detection (with the CellSearch^®^ method) with a ≥ 1 CTC/7.5 mL cutoff (10.5% of patients) was an independent prognostic marker (HR = 5.5; 95%CI 2.3–13.6), while Sotelo *et al*. reported no association after surgery in 519 patients with a ≥ 1 CTC/7.5 mL cutoff (35% of patients, HR = 1.03, *P* = 0.89)[32]. Among patients with high‐risk CRC requiring adjuvant chemotherapy, CTC detection after chemotherapy correlated with a higher relapse rate [32, 33]. In 2017, a meta‐analysis including 20 studies (*n* = 3687 patients) demonstrated that CTC detection in blood (ranging from 8.8% to 74% of patients) was correlated with poorer outcome (reduced disease survival with HR = 2.4; 95% CI 1.7–3.5) [34]).

In locally advanced pancreatic cancer, a prospective study of 79 patients showed that baseline CTC detection was an independent prognostic factor for OS, but the detection rate was surprisingly low in this setting (5% at baseline and 9% 2 months after chemotherapy or chemoradiotherapy) [35]. This low detection rate could be explained by sequestration of CTCs in the liver (an explanation that could also be valid in colorectal cancer) or decreased vascularity of aggressive tumors [36]. These results highlight the fact that CTC levels can vary in different cancers due to anatomical differences.

#### 2.2.3 Lung cancer

The relationship between the presence of CTCs and the risk of relapse was studied in patients with stage I‐IIIa nonsmall cell lung cancer (NSCLC) treated with curative intent. Patients with detectable CTCs before and after surgery had shorter DFS and OS than those without CTCs [37]. This trend was particularly evident in the postoperative period, with a significant prognostic value for DFS [38, 39, 40].

#### 2.2.4 Other cancers

The prognostic impact of CTC detection on DFS or OS has been widely demonstrated in many other cancers in the nonmetastatic setting, such as melanoma [41, 42], head and neck cancer [43], bladder cancer [8], testicular cancer [44], and other cancers. However, few studies have been conducted on localized prostate cancer, with conflicting results [45, 46].

#### 2.2.5 Peripheral blood versus venous blood collection during surgery

While CTCs are generally assessed in peripheral blood samples, CTCs can also be detected during surgery, with interesting results. In pancreatic cancer, patients with CTCs detected in the portal vein had a higher risk of liver metastases after surgery than those with CTCs detected in peripheral blood [47]. In lung cancer, in which the peripheral blood CTC detection rate is low, CTCs were detected in the pulmonary vein (PV) with a CTC detection rate of 43% (range: 1–3093 CTCs/7.5 mL of blood), while the CTC detection rate in peripheral blood was 22% (range: 1–4 CTCs per 7.5 mL) [48]. In the TRACER‐X study, PV‐CTCs were detected in 48% of patients (*n* = 100) and were associated with lung cancer‐specific relapse (*P* = 0.009 log‐rank, HR = 2.78) [49].

In summary, CTCs can be detected in nonmetastatic cancers and are a strong prognostic factor for relapse or death, but the low detection rate with techniques such as CellSearch^®^, and the lack of standardization of time‐points (before or after surgery or neoadjuvant therapy) make implementation of CTC detection in clinical practice challenging in this setting.

### 2.3 Advanced tumors

The studies described below mostly used the CellSearch^®^ system, the only technique that has been approved by the FDA in BC, CRC and prostate cancer [50, 51, 52].

#### 2.3.1 Breast cancer

In the first large study using the CellSearch^®^ system to enumerate CTCs in healthy subjects and in patients with nonmalignant diseases or various metastatic cancers [4], the mean number of CTCs per 7.5 mL of blood for BC patients was 84 (standard deviation: ±885), and 26% of BC patients had ≥ 5 CTCs/7.5 mL.

In a seminal prospective study conducted in 20 centers, Cristofanilli *et al*. studied the prognostic value of CTCs in 177 patients with metastatic BC [50, 53]. CTC counts were performed at two time‐points: before starting a new treatment, and three to four weeks after initiation of treatment. In a training set of 102 patients, the authors determined that the optimal cutoff for defining patients with high CTC counts with the first blood draw was ≥ 5 CTCs per 7.5 mL, a higher cutoff than the ≥ 1 CTC cutoff commonly used for localized BC. None of the subjects of a control cohort without cancer had a ≥ 5 CTC count, while at least five CTCs were detected in 49% of patients with metastatic BC. Median PFS and median OS were significantly lower in patients with high CTC counts. Similar results were observed at the second CTC evaluation, after starting treatment. In a multivariate Cox model, CTC counts (at baseline or at 3–4 weeks) were independent predictors of PFS, even when focusing on patients in the first‐line setting [53].

A pooled analysis (individual‐based) of 1,944 patients with metastatic BC from 17 centers demonstrated that a baseline CTC count of ≥ 5 per 7.5 mL was associated with decreased PFS and OS in a multivariate model (HR = 1.9; 95%CI 1.7–2.1 and HR = 2.8, 95%CI 2.4–3.2, respectively) [54]. When building a prognostic model for OS and PFS with clinicopathological characteristics independently associated with OS or PFS in the multivariate model, adding CTC status enhanced the model, and was superior to other blood biomarkers (CEA—carcinoembryonic antigen—and CA 15‐3).

Using CTC monitoring during treatment in addition to the baseline CTC assessment provided additional information in both the study by Cristofanilli *et al*. [50] and the pooled analysis (CTC assessment at 3–5 weeks and 6–8 weeks) [54]. The OS and PFS of patients with < 5 CTCs both at baseline and after starting therapy did not differ significantly from the OS and PFS rates of patients with ≥ 5 CTCs at baseline and < 5 CTCs after therapy. Similarly, the OS and PFS of patients with ≥ 5 CTCs both at baseline and on therapy were not significantly different from the OS and PFS of patients with < 5 CTCs at baseline and ≥ 5 CTCs after starting therapy. Interpretation of CTC variations was studied in more detail in the observational phase of the CirCe01 trial [55]: CTCs were assessed in patients about to start a third line of treatment for metastatic BC, and participants with at least 1 CTC/7.5 mL at baseline underwent a second CTC evaluation before starting their second cycle of chemotherapy. This study defined a cutoff for the CTC response: patients with ≥ 5 CTC/7.5 mL at baseline and a relative decrease of at least 70% of the CTC count or with < 5 CTC/7.5 mL at the second evaluation had a significantly higher PFS. A recent study (469 patients −2,202 samples) found that the CTC trajectory (tCTC) patterns during the course of treatment was a better predictor of PFS and OS compared to baseline CTC and combined CTC (baseline—end of cycle 1) models [56].

These studies highlight that 1) CTC monitoring has a higher prognostic value than a simple baseline CTC count 2) the decrease rate of CTC count, and not only the numbers of CTCs, is required to assess prognosis of cancer.

#### 2.3.2 Gastrointestinal cancers

In metastatic CRC, a meta‐analysis (publication‐based) including 11 studies (*n* = 1847 patients) showed that an elevated CellSearch^®^‐based baseline CTC count (using various cutoffs ranging from 1 to 5 CTCs/7.5 mL) was a strong and independent prognostic factor for PFS (HR = 1.8; 95% CI 1.5–2.1) and OS (HR = 2; 95% CI 1.5;2.7) [57]. Other studies have found similar results [58, 59]. In a phase II study, Krebs *et al*. [60] found that patients with a high baseline CTC count (≥3 CTCs/7.5 mL) could benefit from an intensive chemotherapy regimen (four drugs), unlike patients with low CTC counts. This study suggests that the baseline CTC count could help to select patients eligible for intensification of chemotherapy, and a clinical trial based on this principle is described below.

The PRODIGE 17 trial, conducted in patients with advanced gastric and esophageal cancer, reported that dynamic changes in CTC count between baseline and 4 weeks after treatment were significantly associated with PFS and OS [61].

#### 2.3.3 Prostate cancer

Many studies have investigated the prognostic value of the baseline CTC count in metastatic prostate cancer. Using a cutoff value of ≥ 5 CTCs per 7.5 mL of blood, the CTC count was reversely associated with OS [52, 62, 63, 64]. In a large‐scale prospective study, Sher *et al*. evaluated the potential of CTC count as a surrogate biomarker of treatment efficacy in 711 patients with metastatic castration‐resistant prostate cancer (CRPC) pretreated by docetaxel. A CTC count of ≥ 5 CTC/7.5 mL after 12 weeks of treatment was associated with poor survival. Moreover, the combination of CTC count and lactate dehydrogenase (LDH) was confirmed as a surrogate marker for OS: the 2‐year OS was 46% in the low‐risk group (CTC < 5/7.5 mL and LDH < 250 U/L) versus 2% in the high‐risk group (≥5 CTCs/7.5 mL of blood and LDH > 250 U/L) [65, 66].

#### 2.3.4 Lung cancer

The CTC count has also been shown to be an independent prognostic factor in metastatic lung cancer. Hou *et al*. found that patients with small cell lung cancer (SCLC) and a CTC count of ≥ 50 CTCs/7.5 mL (reflecting the very high CTC counts observed in SCLC) prior to chemotherapy had a poorer clinical outcome in terms of OS than those with a CTC count < 50 CTC/7.5 mL (HR = 2.5; 95% CI 1.4–4.3, *P* = 0.002)[67]. A recent European multicenter study in 550 patients with NSCLC using the CellSearch^®^ system reported that 27% of patients had ≥ 2 CTCs and 13% had ≥ 5 per 7.5 mL of blood [68]. CTC counts of ≥ 2 and ≥ 5 per 7.5 mL were associated with reduced PFS (≥2 CTCs: HR = 1.7, ≥5 CTCs: HR = 2.2, both *P* < 0.001) and OS (≥2 CTCs: HR = 2.2, ≥5 CTCs: HR = 2.8, both *P* < 0.001), in agreement with other studies [69, 70].

In summary, the CTC detection rate and the CTC count are higher in the metastatic setting than in the nonmetastatic setting and are strongly associated with patient outcome, with a CellSearch^®^ cutoff to define poor prognosis of about 3–5 CTC/7.5 mL (with the exception of SCLC). Variations in CTC count during treatment may also be a biomarker of treatment efficacy. In BC, CTC monitoring during treatment is a more useful marker than simple baseline CTC level, as patients with a high baseline CTC count have a good prognosis when an early decrease of CTCs is observed.

### 2.4 Molecular characterization of CTCs

One of the main concepts investigated in clinical trials concerns the use of CTC as a surrogate for tumor material. Cancer diagnosis and analysis of the main tumor genomic alterations and gene and protein expression are currently performed on tumor biopsy, with the exception of genomic alterations, which can now be detected by ctDNA [71, 72]. Using CTCs from the patient's peripheral blood could be an attractive noninvasive alternative to assess molecular tumor characteristics, tumor heterogeneity and variations during treatment.

#### 2.4.1 Theranostic detection of genomic alterations in CTC

Theranostic detection of actionable genomic alterations, such as *EGFR* or *ALK*, widely described with ctDNA [73], is feasible on CTC [74]. For example, in one study isolated CTCs from patients with metastatic NSCLC were screened for *EGFR‐*activating mutation using both standard nucleotide sequencing and the Scorpion Amplification Refractory Mutation System (SARMS) technology (DxS) (the assay combined two technical methods, Scorpion and ARMS, to detect mutations in real‐time PCRs). This study identified the *EGFR* mutation in the CTCs of 11 out of 12 patients (92%) and in matched free plasma DNA from 4 out of 12 patients (33%) (*P* = 0.009). Mutational analysis of CTCs at disease progression could detect new *EGFR* mutations (including the T790 mutation) [75].

Circulating tumor cell analysis can also identify genetic alterations other than gene mutations, including *ALK/ROS1* rearrangements in NSCLC. For example, the numbers of CTCs with *ALK‐* or *ROS1*‐rearrangements, as detected using a fluorescent in situ hybridization (FISH) method on filters (filter‐adapted FISH), changed during treatment with crizotinib [76, 77] and an increase of *ROS1*‐rearranged CTCs was associated with crizotinib resistance [76].

#### 2.4.2 Breast cancer

Several studies have demonstrated the feasibility of determining the HER2 status of CTCs in BC [78, 79, 80], using CellSearch^®^ [81]. For example, the prospective study conducted by Fehm *et al*. [82] included 254 patients with metastatic BC, in whom CTCs were detected by CellSearch^®^ or AdnaTest^®^ BreastCancer (another positive selection‐based method using magnetically coupled antibodies, and allowing for mRNA analysis by reverse transcriptase PCR in a second step). The HER2 status of CTCs was assessed at the transcript level by PCR for the AdnaTest^®^ and at the protein level by immunofluorescence for the CellSearch^®^ system. CTCs were classified as HER2‐positive if a PCR fragment of the *HER2* transcript was detected at a peak concentration of 15 ng μL^−1^ or more. A limited correlation was observed between the HER2 status of CTCs detected by CellSearch^®^ or AdnaTest^®^ and the HER2 status of the primary tumor (e.g., with CellSearch^®^, 33% of patients with a HER2‐negative tumor had HER2‐positive CTCs and 42% of patients with a HER2‐positive tumor had exclusively HER2‐negative CTCs). The value of the HER2 status of CTCs as a surrogate of the HER2 status of the primary tumor classically determined by histopathological methods therefore appears to be limited. However, these results could reflect tumor heterogeneity, as discordant HER2 status between the primary tumor and metastases has been described, although with lower rates [83].

The molecular characterization of CTCs in BC has also been used to detect mutations in genes of interest. For instance, several studies have investigated *PIK3CA* mutations in CTCs [84, 85, 86], as some of these mutations now have a theranostic value in metastatic BC [87]. In a recent study [86], *PIK3CA* hotspot mutations were assessed in CTCs (using the CellSearch^®^ system) and paired ctDNA, in 56 samples from 43 patients with early BC and in 27 samples from 16 patients with metastatic BC. Corresponding primary tumor samples from 16 of these patients were also analyzed. CTCs and leukocytes were recovered from CellSearch^®^ cartridges after CTC enumeration; genomic DNA extraction and real‐time PCR were used to detect the E545K and H1047R *PIK3CA* hotspot mutations. These mutations were detected in 37/56 (66%) of CellSearch^®^ cartridges from early BC patients and in 23/27 (85%) of CellSearch^®^ cartridges from metastatic BC patients. The concordance between these results and paired ctDNA samples was low (48% for early BC, 7% for metastatic BC). In addition, it should be noted that no CTCs were actually detected by the CellSearch^®^ system in many of the samples for which the CellSearch^®^ cartridge was tested positive for a *PIK3CA* mutation, possibly due to the presence of EpCAM‐positive, but cytokeratin‐negative CTCs in the cartridge. Other methods have therefore been developed, notably based on the isolation and analysis of single CTCs [85].

#### 2.4.3 Prostate cancer

In prostate cancer, the androgen receptor (AR) pathway is one of the most important pathways implicated in disease progression and resistance to treatment and is therefore targeted by androgen deprivation therapy and hormone therapy. A splice variant of AR, namely AR‐V7, has been linked to resistance to secondary hormonal agents such as enzalutamide and abiraterone [88], and patients with AR‐V7‐positive CTCs (AR‐V7(+) CTCs) had a better outcome when treated by a taxane [89] (AR‐V7 is discussed in more detail in the clinical utility section).

A transmembrane protein overexpressed in most prostate cancers, prostate‐specific membrane antigen (PSMA), has recently been studied for both diagnostic and therapeutic applications [90]. This protein has been associated with higher Gleason score, tumor stage and biochemical relapse [91]. Marked heterogeneity of PSMA expression in CTCs has been reported among patients with prostate cancer, and a number of discrepancies have been reported between PSMA expression on tumor tissue and CTC in the same patient. This difference could explain the lack of response of these patients to PSMA‐targeted therapies [92].

A phase 2 trial evaluated the safety and efficacy of BIND‐014, a docetaxel nanoparticle targeting PSMA, in combination with prednisone, and in addition to the primary endpoint (PFS), changes in CTC count based on PSMA expression levels on CTCs were evaluated by EPIC Sciences technology. While CTCs were detected in 16 of 18 patients (89%), not all patients had PSMA‐positive CTCs (PSMA(+) CTCs), as only 11 of 18 (61%) patients had PSMA(+) CTCs at baseline. Interestingly, after treatment, PSMA(+) CTCs were preferentially reduced [93]. The presence of PSMA(+) CTCs could therefore be used as a biomarker to select patients for PSMA‐based treatment, and PSMA(+) CTCs could be used to monitor the efficacy of these treatments. However, larger studies are needed to confirm this hypothesis.

#### 2.4.4 PD‐L1 testing

The emergence of immunocheckpoint inhibitors (ICI) such as PD‐1 or PD‐L1 inhibitors has led to interesting results in terms of durable response in certain metastatic cancers [94]. PD‐L1 expression assessed by immunohistochemistry on tumor tissue is currently the major biomarker to predict the efficacy of ICI. Many studies have shown that PD‐L1 expression can be assessed on CTCs [95, 96, 97, 98] and could represent an alternative to PD‐L1 assessment on tissue [99].

In NSCLC, Nicolazzo *et al*. assessed CTC status with CellSearch^®^ and PD‐L1 staining method at baseline, and at 3 and 6 months in patients treated with nivolumab (an anti‐PD‐1 antibody). Patients with PD‐L1‐negative CTCs at 6 months derived a clinical benefit from nivolumab therapy, while patients with PD‐L1‐positive CTCs (PD‐L1(+) CTCs) experienced disease progression. These results suggest that persistence of PD‐L1(+) CTCs might reflect a mechanism of therapy escape [100]. A recent prospective study that evaluated CTC detection (Parsortix liquid biopsy system and PD‐L1 staining method) reported results consistent with those of previous studies, as patients with tumor response to ICI showed decreased or stable levels of PD‐L1(+) CTC, while patients with disease progression showed increased levels of PD‐L1(+) CTC [101].

PD‐L1 characterization on CTCs could therefore be clinically relevant, particularly for the early detection of resistance to immunotherapy, but larger prospective studies are still needed.

## 3 Clinical utility

### 3.1 Studies based on CTC count and/or monitoring

**Figure 1:**
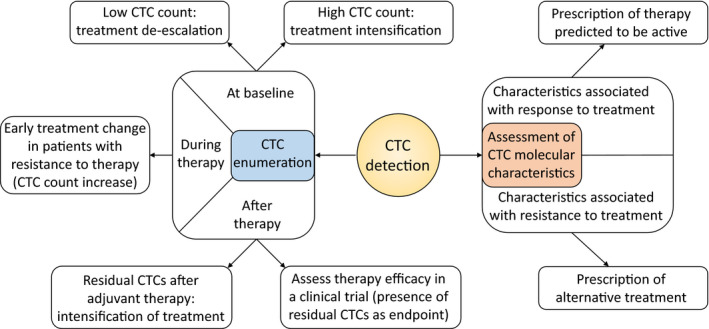
Circulating tumor cells could improve the management of cancers in several ways. Using the prognostic value of the CTC count or of the CTC fluctuations could allow for the treatment intensification in patients with a poor prognosis or de‐escalation in patients with a good prognosis. It could also serve as an endpoint to evaluate the efficacy of a treatment. Molecular characteristics of CTCs could also be used for their theranostic value.

With the growing body of evidence concerning the prognostic value of CTCs, clinicians began to investigate interventions able to increase survival in patients with poor prognosis associated with high CTC count or an unfavorable CTC variation. They also investigated whether the biological information that can be extracted from CTCs could improve patient care. These studies are detailed in Table 1 and Figure 1 summarizes the possible applications of CTCs for clinical management of patients that have been investigated to date.

**Table 1:** Published or ongoing trials assessing the clinical utility of CTCs

Trial name and organ	Primary objective	Results (for published trials)
*Phase III*		
VISNU‐1 NCT01640405 COLON [111]	Compare the efficacy, in terms of PFS, of first‐line triplet chemotherapy (FOLFOXIRI‐bevacizumab) and doublet chemotherapy (FOLFOX‐bevacizumab) in metastatic colorectal cancer patients with elevated baseline CTC count (≥3/7.5mL) with CellSearch^®^ method.	Median PFS was 12.4 months (95% CI 11.2 to 14.0) with FOLFOXIRI bevacizumab and 9.3 months (95% CI 8.5 to 10.7) with FOLFOX‐bevacizumab (stratified HR, 0.64; 95% CI 0.49 to 0.82; p = 0.0006).
SWOG S0500 NCT00382018 BREAST [105]	Compare OS between metastatic BC patients randomly assigned to early change or continuation of first‐line chemotherapy in the presence of persistently high CTC count (≥5/7.5mL using the CellSearch^®^ system) between baseline and assessment after 22 days of treatment	No difference in median OS (10.7 months in standard arm, 12.5 months in experimental arm, p = 0.98)
CirCe01 NCT01349842 BREAST [108]	Compare OS between metastatic BC patients randomly assigned to early change or continuation of ≥ third‐line chemotherapy in the presence of persistently high CTC count (≥5/7.5mL using the CellSearch^®^ system) or < 70% decrease between baseline and the beginning of a second cycle of chemotherapy	Negative
STIC‐CTC NCT01710605 BREAST [109]	Compare PFS (noninferiority design) between HER2‐negative, hormone receptor‐positive metastatic BC patients randomized to a clinician‐driven or a CTC‐driven choice of first‐line treatment (chemotherapy if ≥ 5/7.5mL using the CellSearch^®^ system, endocrine therapy if < 5 CTCs/7.5mL)	The CTC‐driven choice was noninferior to the clinician‐driven choice in terms of PFS: 15.5 months (95%CI, 12.7 to 17.3) in the CTC arm vs. 13.9 months (95%CI, 12.2 to 16.3) in the standard arm
DETECT III NCT01619111 BREAST	Compare the CTC clearance rate of HER2‐negative metastatic BC patients with ≥ 1/7.5mL HER2‐positive CTC (using the CellSearch^®^ system coupled with the evaluation of the HER2 status by IHC or FISH) randomized to standard treatment with or without lapatinib	Ongoing
*Phase II*		
CABA‐V7 NCT03050866 PROSTATE	Evaluate the PSA response to cabazitaxel in mCRPC patients with AR‐V7‐positive CTCs (using CellSearch^®^ method)	Ongoing
PROPHECY NCT02269982 PROSTATE [117]	Validate the prognostic significance of baseline CTC AR‐V7 in patients treated with endocrine therapy (using CellSearch^®^ method coupled with AdnaTest CTC AR‐V7 mRNA and the Epic Sciences protein‐based assay).	Patients with AR‐V7(+) mCRPC had a very poor outcome (median PFS = 3.1 months, 0‐11% of PSA response ≥ 50%) when treated with endocrine therapy
TREAT‐CTC NCT01548677 BREAST [102]	Assess the rate of patients with persistent CTCs after randomization and treatment by 6 cycles of trastuzumab or observation, in subjects with HER2‐negative BC with ≥ 1/15mL (using the CellSearch^®^ system) CTC after surgery and (neo)adjuvant chemotherapy	Stopped for futility: 1,317 patients screened, 63 randomized, 5 had at ≥ 1 CTC at week 18 in the trastuzumab arm vs. 4 in the standard arm
I‐CURE‐1 NCT03213041 BREAST	Evaluate PFS in triple‐negative metastatic breast cancer with ≥ 5 CTCs/7.5mL (using the CellSearch^®^ system) treated by pembrolizumab + carboplatin	Ongoing
LAP105594 NCT00820924 BREAST [112]	Assess the efficacy of lapatinib in patients with metastatic HER2‐negative BC but HER2‐positive CTCs (≥2 CTCs/7.5mL as assessed by the CellSearch^®^ system, with a HER2 status evaluated by immunofluorescence and FISH) who had received at least one line of therapy	Only 7 of the 96 patients with detectable CTCs had HER2 + CTCs. No objective responses were detected.
CirCe T‐DM1 NCT01975142 BREAST [113]	Assess the tumor response rate to trastuzumab‐emtansine in patients with metastatic HER2‐negative BC but *HER2*‐amplified CTCs (at least one *HER2*‐amplified CTC/7.5ml, as determined by CellSearch^®^ and FISH) who had received at least two lines of chemotherapy	Among the 154 screened patients, 11 received trastuzumab‐emtansine and one had a partial response.
NCT01048099 BREAST [132]	Evaluate the clinical significance of the PRO Onc assay and assess the efficacy of HER2‐targeted therapy (trastuzumab/pertuzumab) in patients with HER2‐negative breast cancer identified as having HER2 overexpression/activation by the PRO Onc Assay.	Fourteen patients were treated with HER2‐targeted therapy. Twelve of the 14 patients progressed within 6 weeks, one patient had a brief (12 weeks) partial response, and one patient was stable for 12 weeks.

BC, breast cancer; CTC, circulating tumor cells; FISH, fluorescent in situ hybridization; IHC, immunohistochemistry; mCRPC, metastatic castration‐resistant prostate cancer; OS, overall survival.

#### 3.1.1 Breast cancer

The phase II TREAT‐CTC trial screened 1317 patients with HER2‐negative BC treated with curative intent and included 95 participants with ≥ 1 CTC per 15 mL of blood after surgery and (neo)adjuvant chemotherapy: 63 were randomized to 6 cycles of trastuzumab (anti‐HER2 monoclonal antibody) or observation [102]. The primary endpoint was the percentage of patients with a persistent CTC count ≥ 1 per 15 mL at week 18. However, the trial was stopped for futility: five patients still had at least one CTC at week 18 in the trastuzumab arm versus four in the standard arm. Most patients (76%) had HER2‐negative CTCs. This result can be explained by the lack of efficacy of trastuzumab in HER2‐negative BC. At the time of the study design, it was still unclear whether HER2‐negative BC patients could benefit from trastuzumab treatment [103], but the TREAT‐CTC trial could have predicted the lack of efficacy of adjuvant trastuzumab in HER2‐negative BC (HER2 FISH‐negative with immunohistochemistry (IHC) 1+ or 2+) in the phase III NSABP B‐47/NRG trial [104]. Nevertheless, although the chosen intervention was not effective, the TREAT‐CTC trial demonstrated the feasibility of escalation of adjuvant chemotherapy based on CTC positivity, provided a large number of patients is screened.

As already mentioned, Cristofanilli *et al*. [50] demonstrated that not only CTC levels, but also the fluctuation of CTC numbers over time could identify a population of high‐risk patients with metastatic BC who might benefit from an early change of therapy. On the basis of this observation, the SWOG S0500 randomized trial was designed [105], where 319 of the 595 study participants had ≥ 5 CTCs per 7.5 mL at baseline and were selected for a second CTC assessment 22 days after the start of first‐line therapy for metastatic BC. Patients with persistently high CTCs at second assessment (*n* = 123) were randomly assigned to a change of therapy or continuation of the same therapy. The primary endpoint was OS. Although the prognostic value of CTCs was confirmed, no statistically significant difference was observed between the two randomized arms. Several reasons have been proposed to explain this result: as discussed in one comment [106], if CTC detection techniques were able to identify all patients who developed resistance to first‐line therapy, they would allow early change of treatment up to 2 months before radiological or clinical signs of progression, which could be insufficient to translate into an OS benefit for patients assigned to the experimental arm. Furthermore, the CTC cutoff for interventional trials was not optimized, as shown in the nonrandomized part of the CirCe01 trial [55].

As the utility of CTCs may be greater in a population with a higher prevalence of chemoresistance, the CirCe01 trial (NCT01349842) was designed to evaluate the utility of dynamic CTC changes in response to treatment in a cohort of patients with metastatic BC, starting a third‐line chemotherapy [107]. A total of 265 patients with a baseline CTC count of ≥ 5 per 7.5 mL were included and randomized between a CTC arm and a standard arm. In the CTC arm, another CTC assessment was performed before the start of a second cycle of chemotherapy: if the CTC count decreased by less than 70% and remained at ≥ 5 CTCs [55], the investigator had to propose another line of treatment (with a new CTC assessment). This trial failed to demonstrate that an early switch of chemotherapy improved OS, due to the limited accrual and compliance, although, in a *post hoc* analysis, patients with no CTC response who switched chemotherapy experienced longer survival than patients who did not switch chemotherapy [108].

The STIC‐CTC trial also adopted another approach in metastatic BC [109]: in HER2‐negative, hormone receptor‐positive metastatic BC, first‐line chemotherapy is usually preferred to endocrine therapy in the presence of a ‘visceral crisis’, *that is,* rapidly progressive, life‐threatening disease. However, this remains a poorly defined concept, and the choice of chemotherapy or endocrine therapy is still largely based on the clinician’s assessment. The STIC‐CTC trial compared a clinician‐based choice of first‐line therapy (chemotherapy or endocrine therapy) to a CTC‐based choice (chemotherapy for patients with baseline CTC count of ≥ 5 CTCs/7.5 mL of blood, endocrine therapy for patients with baseline CTC count of < 5 CTCs/7.5 mL of blood). The primary endpoint was the PFS, with a noninferiority design, as the rate of chemotherapy prescription was expected to be lower in the CTC arm, allowing for de‐escalation. A total of 761 patients were randomized to the CTC arm or the clinician choice arm. The study met the noninferiority endpoint, but the rate of chemotherapy prescription was higher in the CTC arm (32% *versus* 27% in the clinician choice arm). Planned subgroup analyses found a PFS benefit of the CTC‐driven choice (experimental arm) for patients over 60 years old. In addition, in the subgroup with ≥ 5 CTCs/7.5 mL but with endocrine therapy as the preferred choice by the investigator, PFS was significantly higher in the CTC‐driven choice arm (in which patients received chemotherapy). In an unplanned analysis, when pooling both groups with a discordant clinician/CTC estimate (≥ 5 CTCs/7.5 mL but endocrine therapy preferred by the investigator; or < 5 CTCs/7.5mL but chemotherapy preferred by the investigator), the PFS and OS were significantly higher in patients treated with chemotherapy.

This was the first trial to suggest that intensity of treatment could be based on CTC count in metastatic BC. However, this trial was conducted before the results of the first‐line phase III trials on CDK4/6 inhibitors [110] and its conclusions cannot be extrapolated to CDK4/6 combination therapy. CTCs are currently being evaluated as a secondary outcome in the recently initiated AMBRE trial (NCT04158362), which compares first‐line chemotherapy to endocrine therapy in combination with the CDK4/6 inhibitor abemaciclib.

CTCs could also be used to select a high‐risk population that could benefit from certain treatment regimens: in metastatic triple‐negative BC, Cristofanilli *et al*. have initiated a phase II trial evaluating the combination of pembrolizumab and carboplatin in patients with ≥ 5 CTCs/7.5 mL at baseline (NCT03213041).

#### 3.1.2 Colorectal cancer

Following the results of the study by Krebs *et al*.[60] indicated above, a randomized phase III clinical trial (NCT01640405) has been initiated in patients with metastatic CRC and a CTC count of ≥ 3/7.5 mL, as determined with CellSearch^®^ at a first‐line setting. This study evaluated whether an intensive four‐drug chemotherapy regimen (FOLFOXIRI + Bevacizumab) achieves a better outcome *versus* a three‐drug regimen (FOLFOX + Bevacizumab) in a high‐risk population, where the regimen was defined by baseline CTC level (Table 1).

Recent results showed that first‐line FOLFOXIRI + bevacizumab significantly improved median PFS compared with FOLFOX + bevacizumab in patients with metastatic CRC and ≥ 3 CTCs at baseline; the values being 12.4 months (95% CI 11.2 to 14.0) and 9.3 months (95% CI 8.5 to 10.7), respectively (stratified HR, 0.64; 95% CI 0.49 to 0.82; *P* = 0.0006)[111].

However, this study does not answer the opposite question, at a time when chemotherapy intensification is becoming the standard of care: can intensification be avoided in low‐risk patients? This study and the STIC‐CTC trial in BC highlight the difficulty of conducting clinical trials based on CTC levels to escalate or de‐escalate systemic treatment in the context of rapidly changing standard of care, as the standard of care has often evolved between initiation of the study and evaluation of the results.

### 3.2 Studies based on molecular characteristics of CTCs

#### 3.2.1 Breast cancer

Since several studies have demonstrated the feasibility of assessing molecular characteristics of CTCs, such as HER2 expression, interventional trials have been designed to evaluate whether the use of these methods could improve patient care.

In an interventional trial conducted by Pestrin *et al*.[112], pretreated patients with HER2‐negative metastatic BC and HER2‐positive CTCs were treated by the HER2‐inhibitor lapatinib (1500 mg/day). HER2‐positivity in CTCs was defined by immunofluorescence as expression of HER2 in at least 50% of CTCs, when HER2 was not amplified or nonevaluable in FISH, or as expression of HER2 in less than 50% of CTCs, when HER2 was found to be amplified by FISH (*HER2* to chromosome 17 centromere signal ratio > 2.2). However, only 7 of the 96 patients with detectable CTCs (≥ 2 CTCs/7.5 mL) had HER2‐positive CTCs; and among those, the best response to lapatinib was one stable disease (8.5 months), while the remaining patients had progressive disease. The CirCe T‐DM1 trial [113] adopted a similar approach, including HER2‐negative metastatic BC patients who had received at least two lines of chemotherapy. Participants had to have CTCs bearing a *HER2* amplification (as assessed by FISH, with a *HER2*/chromosome 17 centromere ratio ≥ 2.2 or > 6 *HER2* copies per cell) to be eligible for the treatment phase. Patients with ≥ 1 CTC/7.5 mL and *HER2*‐amplified CTCs were treated with trastuzumab‐emtansine. Among the 154 screened patients, 14 had at least one *HER2*‐amplified CTC per 7.5 mL and 11 of them received trastuzumab‐emtansine, with a partial response in one patient. These studies illustrate that a relatively small number of patients with HER2‐negative metastatic BC have *HER2*‐amplified or even HER2‐positive CTCs, and, as a result, few patients were treated with anti‐HER2 therapy. Interestingly, in the CirCe T‐DM1 trial, patients with at least 1 *HER2*‐amplified CTC per 7.5 mL had a low *HER2*‐amplified/*HER2*‐negative ratio, suggesting that these *HER2*‐amplified cells reflect a minor subclone, which may explain the lack of efficacy of anti‐HER2 therapy. The results of the DETECT III trial [114], which randomizes patients with HER2‐negative metastatic BC and detectable HER2‐positive CTC to standard treatment or to standard treatment in combination with lapatinib, are therefore eagerly awaited. In this phase III multicenter trial, HER2‐positive CTCs were identified by IHC or FISH after the detection of CTCs by CellSearch^®^. The primary endpoint of the trial is the CTC clearance rate, which may provide interesting insights into the activity of anti‐HER2 agents in these patients. Patients with HER2‐negative CTCs may be eligible for inclusion in other studies of the DETECT program: patients with hormone receptor‐positive BC and an indication for endocrine therapy receive everolimus or ribociclib in combination with endocrine therapy [114] in the DETECT IVa study, whereas those with an indication for chemotherapy or hormone receptor‐negative BC receive eribulin (DETECT IVb). The DETECT program also includes a large number of translational studies [115]: for instance, in DETECT III, HER2‐positive CTCs will be analyzed for the presence of *PIK3CA* mutations. RANK and RANKL expression on CTCs will also be assessed at baseline and during treatment with denosumab, an anti‐RANKL agent indicated in patients with bone metastases.

#### 3.2.2 Prostate cancer

Following the demonstration of AR‐V7 as a very promising biomarker in prostate cancer, Armstrong *et al*. conducted a prospective study to validate the utility of this biomarker to predict treatment efficacy. They showed that patients with AR‐V7(+) metastatic CRPC had a very poor outcome (median PFS = 3.1 months, 0‐11% of PSA response ≥ 50%) when treated with endocrine therapy. The presence of AR‐V7(+) CTCs (as detected at both the mRNA level by AdnaTest and protein level by an Epic Sciences assay) may therefore guide oncologists to prescribe chemotherapy to patients with CRPC [116, 117]. An ongoing phase II study aims to determine the response of patients with metastatic CRPC and AR‐V7(+) CTCs (as determined by the CellSearch^®^ system) to cabazitaxel (NCT03050866) (Table 1). CTCs are more reliable than PSA levels, which are affected by modulations in androgen receptor signaling [118], and highly sensitive assays for prostate CTC‐derived transcripts have been developed to guide therapies, especially in patients with advanced disease and bone metastases that cannot be easily biopsied [118]. One of these assays is now commercially available in the US, Oncotype DX AR‐V7 Nucleus Detect® test.

## 4 New research for clinical applications of CTCs

### 4.1 Single CTC analysis

**Figure 2:**
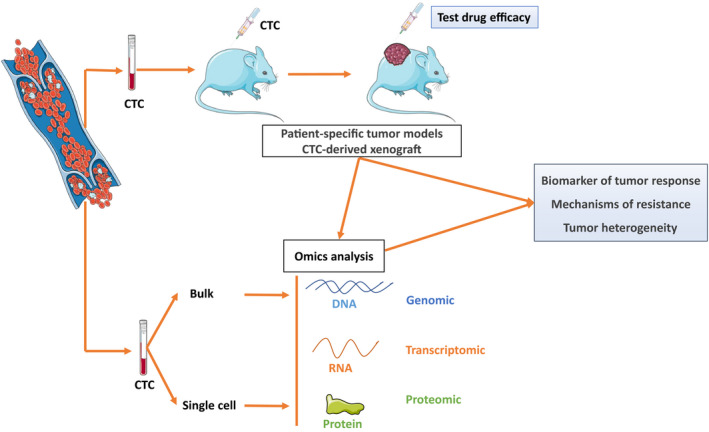
Future application for CTCs. Culture of CTCs could assist in real‐time treatment testing, and CTCs from a patient may be isolated and grafted into mice as CTC‐derived xenografts (CDX), generating a patient‐specific model for drug testing, identification of resistance mechanisms and biomarker development. Genomic, transcriptomic and proteomic analysis of single/bulk CTCs allows evaluation of tumor heterogeneity and evolution during treatment.

Single‐cell analysis is a promising field in oncology and is feasible on CTCs, allowing for several analyses that cannot be performed with ctDNA, such as transcriptomic analysis (Fig. 2). Genomic instability and environmental conditions promote the survival and clonal growth of distinct tumor cell subpopulations, leading to tumor heterogeneity. In addition, while cells sensitive to treatment are destroyed, a subpopulation of cells survive and contribute to resistance to treatment [119, 120]. This tumor resistance is therefore partly due to intratumor heterogeneity (ITH) [121].

Single CTC analysis may help dissect ITH in various cancers. For example, Jordan *et al*. studied the heterogeneity of HER2‐positive CTCs in patients with hormone receptor‐positive, HER2‐negative metastatic BC [122]. In their cohort of 19 patients, 84% had CTCs expressing HER2. HER2‐positive CTCs were more proliferative, but HER2‐negative CTCs showed resistance to chemotherapy. Interestingly, cultured HER2 + and HER2‐ CTCs spontaneously interconverted their phenotype. Importantly, HER2‐positive CTCs did not display any oncogenic addiction, and the HER2‐inhibitor lapatinib was not more active on HER2‐positive CTCs than on HER2‐negative CTCs. Taken together, these results could explain the lack of efficacy of anti‐HER2 agents administered in clinical trials to patients with HER2‐negative BC but HER2‐positive CTCs [112, 113].

More recently, SCLC ITH was studied using chemosensitive and chemoresistant CTC‐derived xenografts (CDX) and analyzing patient CTCs with single‐cell RNA sequencing. This study showed that ITH increased with treatment resistance, involving heterogeneous expression of therapeutic targets and of multiple potential resistance pathways [123].

### 4.2 Culture of CTCs

Interestingly, CTCs derived from a patient can be isolated and grafted into mice as CDX, and provide patient‐specific tumor models for therapy testing and detection of drug resistance mechanisms [124]. These applications have been studied in various cancers, such as BC, prostate cancer, CRC, SCLC [74, 125, 126, 127, 128, 129]. For example, Yu *et al*. [130] derived CDX models from CTCs of BC patients, and DNA sequencing of the CTC lines revealed *de novo* acquired targetable mutations. The authors then tested cell lines for sensitivity to various single drugs and drug combinations targeting the acquired mutations. This strategy highlights the importance of monitoring evolution of the mutation profile throughout the course of the disease.

CTCs from patients with SCLC are tumorigenic in immune‐compromised mice, and CDXs recapitulated the genomic profile of CellSearch^®^‐enriched CTCs, and mimicked the donor patient’s response to platinum and etoposide chemotherapy, proving the clinical relevance of these models. Moreover, genomic analysis of isolated CTCs revealed considerable similarity with the corresponding CDX [131]. Other CDX models have been described in NSCLC [74], melanoma and prostate cancer [128].

Culture of patient‐derived CTCs and the possibility of generating CDXs therefore constitute new tools for drug development, to further our understanding of drug resistance, to explore the biology of advanced cancers and to identify novel biomarker signatures.

## 5 Conclusions and perspectives

Over the last two decades, the prognostic value of CTCs has been clearly demonstrated in the most common tumor types, in both localized and metastatic settings. However, oncologists are just beginning to understand how to use the information derived from CTC detection and analysis for patient care. To achieve this goal, clinicians and researchers will have to address the main limitations of CTCs in clinical practice: the low detection rate with current techniques in the screening or adjuvant settings; lack of effective options when CTCs identify resistance to therapy in the metastatic setting; and a limited understanding of the theranostic information provided by molecular analysis of CTCs, which may be different from that provided by pathology‐based investigations.

## Conflicts of interest

FCB and JYP report research grants from Menarini Silicon Biosystems and FCB reports travel support from Menarini Silicon Biosystems. Other authors declare no conflicts of interest.

## Author contribution

LC, FCB, and JYP conceived and designed the review, and AV, NK, and LC wrote the paper.

### Peer Review

The peer review history for this article is available at https://publons.com/publon/10.1002/1878‐0261.12869.
